# Over-expression of lysophosphatidic acid receptor-2 in human invasive ductal carcinoma

**DOI:** 10.1186/bcr935

**Published:** 2004-09-22

**Authors:** Joji Kitayama, Dai Shida, Akihiro Sako, Makoto Ishikawa, Kotaro Hama, Junken Aoki, Hiroyuki Arai, Hirokazu Nagawa

**Affiliations:** 1 Department of Surgical Oncology, The University of Tokyo, Tokyo, Japan; 2 Department of Pharmaceutical Science, The University of Tokyo, Tokyo, Japan

**Keywords:** carcinogenesis, immunohistochemistry, lysophosphatidic acid, RT-PCR

## Abstract

**Introduction:**

Lysophosphatidic acid (LPA) is a bioactive phospholipid with diverse effects on various cells. It interacts with at least three G-protein-coupled transmembrane receptors, namely LPA1, LPA2 and LPA3, whose expression in various tumours has not been fully characterized. In the present study we characterized the expression profile of LPA receptors in human breast cancer tissue and assessed the possible roles of each receptor.

**Methods:**

The relative expression levels of each receptor's mRNA against β-actin mRNA was examined in surgically resected invasive ductal carcinomas and normal gland tissue using real-time RT-PCR. LPA2 expression was also examined immunohistochemically using a rat anti-LPA2 monoclonal antibody.

**Results:**

In 25 cases normal and cancer tissue contained LPA1 mRNA at similar levels, whereas the expression level of LPA2 mRNA was significantly increased in cancer tissue as compared with its normal counterpart (3479.0 ± 426.6 versus 1287.3 ± 466.8; *P *< 0.05). LPA3 was weakly expressed in both cancer and normal gland tissue. In 48 (57%) out of 84 cases, enhanced expression of LPA2 protein was confirmed in carcinoma cells as compared with normal mammary epithelium by immunohistochemistry. Over-expression of LPA2 was detected in 17 (45%) out of 38 premenopausal women, as compared with 31 (67%) out of 46 postmenopausal women, and the difference was statistically significant (*P *< 0.05).

**Conclusion:**

These findings suggest that upregulation of LPA2 may play a role in carcinogenesis, particularly in postmenopausal breast cancer.

## Introduction

Lysophosphatidic acid (LPA; 1- or 2-acyl-*sn*-glycero-3-phosphate), the simplest glycerophospholipid, mediates a broad range of cellular responses including smooth muscle cell contraction, platelet aggregation, neurite retraction/cell rounding, regulation of cell proliferation, protection from apoptosis, modulation of chemotaxis and transcellular migration [1-3]. Previous reports have also demonstrated that LPA stimulates proliferation, migration and production of matrix metalloproteinases and angiogenic factors in ovarian cancer cell lines [4-9]. Moreover, a high level of LPA has been detected in ascitic fluid from ovarian cancer patients [4,10,11]. These findings suggest that LPA may be a mediator of tumour development and progression [12].

At least three receptors – LPA1/Edg-2, LPA2/Edg-4 and LPA3/Edg-7 – have been identified as specific receptors for LPA [13,14]. These LPA receptors differ with respect to their distribution in various tissues. LPA1 is broadly expressed in various normal tissues, whereas expression levels of LPA2 and LPA3 are more restricted, which may account for the various biological effects of LPA [13,14]. In recent studies LPA1 and LPA2 were found to have redundant functions in mediating multiple endogenous LPA responses, including phospholipase C activation, calcium mobilization, cell proliferation and stress fibre formation in mouse embryonic fibroblast cells [15,16]. However, in the same study the different phenotypes of knockout mice for each receptor suggested distinct roles for LPA1 and LPA2 *in vivo*.

Previous studies also found that malignant transformation resulted in aberrant expression of LPA2 or LPA3 in ovarian or thyroid cancers, suggesting that LPA may play a role in tumour biology and that shifts in LPA receptor expression are related to carcinogenesis [17-20]. However, patterns of expression of these LPA receptors in other tumours have not been adequately examined. In the present study we characterized the expression patterns of LPA receptors in human breast cancer tissue (another common form of cancer in females) and analyzed correlations with other clinical and pathological findings to determine the possible role played by each LPA receptor in the development of breast cancer.

## Methods

### Patients and materials

In the first part of the study, mRNA expression for each LPA receptor was evaluated in 25 cases of invasive ductal carcinoma, which were surgically resected in the Department of Surgery, University of Tokyo, from 1998 to 2003, and in six samples of adjacent normal gland tissue. In the next part of the study, protein expression of LPA2 was evaluated by immunohistochemical staining in the 25 cases and an additional 59 cases of invasive ductal carcinoma, which were resected from 1992 to 1997, also in the Department of Surgery, University of Tokyo.

All of the resected primary tumours and regional lymph nodes were histologically examined by haematoxylin–eosin staining, in accordance with the International Union Against Cancer TNM classification. Several discrete histological parameters and lymph node metastasis were also examined. Oestrogen receptor levels were evaluated using enzyme immunoassay of frozen tumour specimens with cutoff levels for positivity of 5 fmol/mg protein.

### Isolation of total RNA and reverse transcription

The tumour tissue resected from the primary lesion and paired nontumour tissue (taken 10 cm away from the neoplasm) were immediately frozen in liquid nitrogen and kept at -80°C until extraction of RNA. Total RNA was extracted from each sample using the acid guanidine isothiocyanate/phenol/chloroform extraction method. One microgram of total RNA was reverse transcribed using a SuperScript First-Strand Synthesis System (Invitrogen Co., Carlsbad, CA, USA). The reverse transcription reaction was carried out in a total volume of 20 μl, in accordance with the manufacturer's instructions. The cDNA was stored at -20°C until use.

### Preparation of cDNA calibrators

We used pcDNA3 vector (Invitrogen) containing LPA1, LPA2, or LPA3 as the standard cDNA for each LPA receptor [14]. cDNA for human β-actin was prepared using human colon cDNA as the template DNA and the following oligonucleotides: CCATCGAATTCACCACCATGGATGATGATATCGCCGCGCTC and AAGGTGCGGCCGCCTAGAAGCATTTGCGGTGGACGAT. The resulting DNA fragments were digested by *Eco*RI/*Not*I and ligated into pBlueScript (Strategene, La Jolla, CA, USA). The DNA sequence of cDNA prepared by RT-PCR was confirmed by DNA sequencing and used to calibrate for β-actin.

### Real-time fluorescence quantitative PCR

The primers used in the analysis of LPA1, LPA2, LPA3 and β-actin gene expression are given in Table 1. All the primers were designed using Primer Express software (Applied Biosystems, Foster, CA, USA). Real-time PCR reactions were conducted in an ABI PRISM 7000 (Perkin-Elmer/Applied Biosystems, Foster, CA, USA) using SYBR Green I (Perkin-Elmer) with the following profile: one step at 50°C for 2 min, one step at 95°C for 10 min, and 40 cycles at 95°C for 30 s and 60°C for 1 min. Thermocycling was done in a final volume of 20 μl containing 1 μl of cDNA sample. The ABI PRISM software constructed the calibration curve by plotting the crossing point against the logarithm of the number of copies for each calibrator. The number of copies in unknown samples was calculated by comparing their crossing points with the calibration curve. To correct for differences in both RNA quality and quantity between samples, data were normalized using the ratio of the target cDNA concentration to that of β-actin. Both RT and PCR reactions were performed in triplicate, and the mean values were calculated against against β-actin.

### Monoclonal antibodies against human LPA2

A peptide consisting of the carboxyl-terminal 17 amino acids of human LPA2 (335–351) was conjugated with keyhole limpet haemocyanin. The conjugate was injected into the hind foot pads of WKY/Izm rats using Freund's complete adjuvant. The enlarged medial iliac lymph nodes from the rats were used for cell fusion with mouse myeloma cell line PAI. The antibody-secreting hybridoma cells were selected by screening with enzyme-linked immunosorbent assay, immunofluorescence and Western blotting. Several clones were established. In this study, monoclonal antibody from clone 10D5 (rat IgG_1_) was used for immunohistochemical staining.

**Figure 1:**
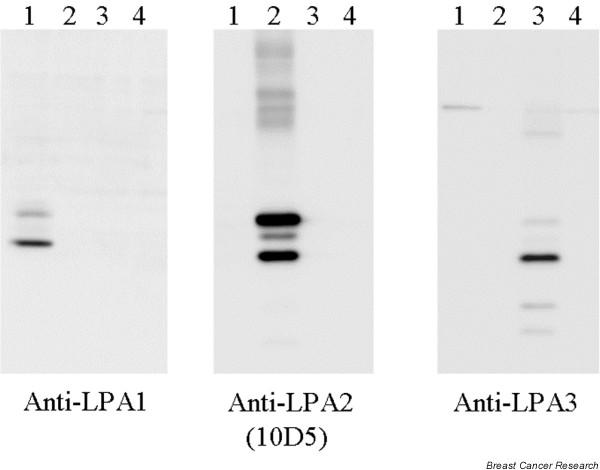
Specificity of anti-human lysophosphatidic acid receptor 2 (LPA2) antibody. The cross-reactivity of anti-human LPA2 (clone 10D5) was examined by Western blotting. Cell lysate of HeLa cells transfected with human lysophosphatidic acid receptor 1 (LPA1; lane1), LPA2 (lane 2), LPA3 (lane 3), or empty vector (lane4) were loaded onto SDS-PAGE. After the proteins were transferred onto nitrocellulose membrane, the LPA2 protein was detected using antihuman LPA2 antibody (clone 10D5) in an enhanced chemiluminescence (ECL) system. 10D5 did not exhibit cross-reactivity to LPA1 or LPA3.

Specificity of the monoclonal antibody against human LPA_2 _used in the present study (clone 10D5) was confirmed as follows. HeLa cells were transfected with LPA_1_-pcDNA3, LPA_2_-pcDNA3, LPA_3_-pcDNA3, or empty pcDNA3 vector using lipofectamine 2000 (Invitrogen). Twenty-four hours after transfection, the cells were rinsed with phosphate-buffered saline (PBS) and recovered using a cell scraper. Protein (10 μg) was separated on SDS-PAGE (12.5% polyacrylamide) and transferred onto nitrocellulose transfer membrane (Schleicher & Schuell, Einbeck, Germany). Using the enhanced chemiluminescence (ECL) method, the expression of each receptor was confirmed using anti-LPA1, anti-LPA2, or anti-LPA3 antibodies (Fig. 1).

### Immunohistochemical study of LPA2

Expression of LPA2 was examined by immunohistochemical staining using the anti-LPA2 antibody. Sections (3 μm thick) were deparaffinized in xylene, hydrated through a gradually diluted ethanol series, and heated in a microwave oven for two 7-min cycles (500 W). After being rinsed in PBS, endogenous peroxidase activity was inhibited by incubation with 0.3% hydrogen peroxide in 100% methanol for 30 min. After washing three times in PBS, nonspecific reaction was blocked by incubation with PBS containing 5% skimmed milk for 30 min at room temperature, and then the sections were incubated with normal rabbit serum for 30 min. The sections were incubated overnight at 4°C in humid chambers with the primary antibody to LPA2 at a dilution of 1/100. As negative control, anti-LPA1 monoclonal antibody (rat IgG_1_), as well as control rat IgG (Nichirei, Tokyo, Japan), was used. After washing three times with PBS, the sections were incubated with biotinylated rabbit antirat immunoglobulin for 30 min. After washing again with PBS, the slides were treated with peroxidase-conjugated streptavidin for 30 min, and developed by immersion in 0.01% hydrogen peroxide and 0.05% diaminobenzidine tetrahydrochloride for 3 min, followed by light counterstaining with Mayer's haematoxylin. Immunoreactivity was assessed by two evaluators who had no knowledge of the background features. When more than half of the carcinoma cells in these fields exhibited significantly stronger staining intensity than the corresponding epithelium from normal mammary glands, these tumours were defined as having high expression of LPA2. In other tumours, most of the carcinoma cells exhibited only weak, if any, immunostaining, and, if present, the staining intensity was similar to that of normal epithelium; these tumours were thus defined as having low expression of LPA2.

### Statistical analysis

All statistical calculations were conducted using StatView-J 5.0 statistical software (SAS Institute, Cary, NC, USA). mRNA levels were compared using unpaired Student's t-test, and relationships between the over-expression of LPA2 and clinicopathological features were examined by Fisher's exact test. *P *< 0.05 was considered statistically significant.

## Results

### Expression of LPA receptors at the mRNA level

**Figure 2:**
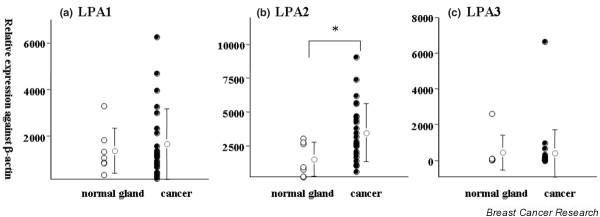
Relative expression level of lysophosphatidic acid receptors' mRNA against β-actin in cancer tissue and normal mammary gland tissue, evaluated using real-time RT-PCR: **(a) **Lysophosphatidic acid receptor 1 (LPA1), **(b) **LPA2 and **(c) **LPA3. Y axis indicates the cDNA copies for lysophosphatidic acid receptors divided by that of β-actin in each case. *P < 0.05.

The relative expression level of each LPA receptor's mRNA was quantitatively evaluated by real-time RT-PCR against that of β-actin (Fig. 2). A significant level of LPA1 mRNA was detected in both normal and carcinoma tissues, and expression levels did not differ significantly between tissues (1438.5 ± 425.5 versus 1675.3 ± 299.8). However, the expression level of LPA2 in cancer tissue was significantly greater than that in normal tissue (3479.0 ± 426.6 versus 1287.3 ± 466.8; *P *< 0.05). In comparison, LPA3 was weakly expressed in both normal and cancer tissue, although one cancer contained a relatively high level of LPA3 transcript. When the ratio of LPA2 to LPA1 was calculated, cancer tissue exhibited a threefold higher ratio than did normal gland tissue (3.69 ± 0.96 versus 1.12 ± 0.43; *P *= 0.0016).

### Expression of LPA2 receptor in the immunohistochemical study

**Figure 3:**
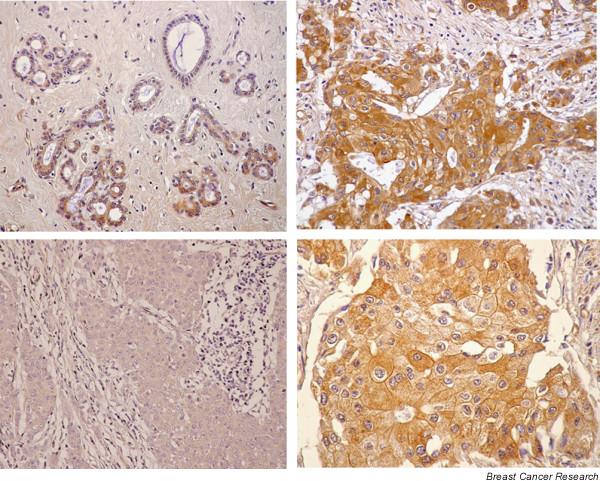
Staining patterns of lysophosphatidic acid receptor 2 (LPA2) in normal mammary epithelium (upper left) and carcinoma tissues (upper right) of a representative case with high expression, and in another tumour tissue sample with low expression of LPA2 (lower left). Some of the normal epithelial cells in the same specimens, as well as interstitial cells, are slightly stained, but the intensity is significantly weaker than that in carcinoma cells with high expression of LPA2. LPA2 can be detected in the cytoplasm as well as in the cell membrane, but not in the nucleus (lower right).

The expression of LPA2 in the same tumours was also evaluated at the protein level by immunohistochemical staining (Fig. 3). In 15 out of 25 (60%) carcinomas, enhanced staining for LPA2 was clearly detected in comparison with normal tissue. In these carcinoma cells, LPA2 was detectable in the cytoplasm and in the cell membrane, but not in the nucleus. Some of the normal epithelium in the same specimens, as well as interstitial cells, also stained slightly, but the intensity was significantly weaker than that in carcinoma cells in the 15 tumours. Thus, these cases were categorized as tumours with high LPA2 expression. In the other 10 tumours, carcinoma cells exhibited apparently the same staining intensity as normal epithelium and were categorized as having low expression of LPA2.

When the immunoreactivity was compared with the relative ratio of LPA2 mRNA against β-actin, 11 out of the 12 tumours (92%) with ratios greater than 3000 had high expression of LPA2, whereas 10 out of 13 tumours with a ratio below 3000 had low expression (Table 1). Thus, the results of the immunohistochemical study exhibited significant correlation with the results of quantitative mRNA study in breast cancer tissue (*P *< 0.001).

### Relationship between LPA2 expression and clinical and pathological factors

Because LPA2 expression showed a strong correlation between mRNA and protein levels in 25 cases, we performed immunostaining of LPA2 in an additional 59 cases. The relations between LPA2 expression and clinical or pathological findings in these 84 cases are presented in Table 2. High expression of LPA2 was frequently detected in relatively old postmenopausal patients. High expression of LPA2 was detected in 17 out of 38 (45%) premenopausal patients and in 31 out of 46 (67%) postmenopausal women; this difference was statistically significant (*P *< 0.05). LPA2 expression did not correlate with oestrogen receptor expression. The frequency of nodal or distant metastasis tended to be higher in tumours with low expression of LPA2, although the difference was not statistically significant.

## Discussion

In the present study we found that breast cancer frequently exhibited enhanced expression of LPA2 mRNA as compared with normal breast gland tissue, although the expression level of LPA1 and that of LPA3 were not significantly different from those in normal tissue. Over-expression of LPA2 was confirmed at the protein level in some of these cases by immunohistochemical staining. Previous studies showed that LPA1 is widely expressed in various tissues, whereas LPA2 and LPA3 are known to be highly expressed in malignant cells, suggesting the potential role of these receptors in the pathophysiology of cancer [5,18,19,21,22]. Our findings regarding LPA2 are basically consistent with those results and suggest that upregulation of the LPA2 gene is a common feature of carcinogenesis in various organs.

Protein expression of LPA2 in various organs has not been investigated thus far because of the lack of an appropriate antibody against LPA2. Our antibody clearly detected significantly enhanced expression of LPA2 in more than half of the ductal carcinoma cells, and the staining pattern exhibited a strong correlation with the mRNA data obtained with quantitative RT-PCR. Normal epithelium of mammary glands also showed slight immunoreactivity, but the staining intensity was markedly weaker than that of carcinoma cells in these cases. Positive staining was mostly detected in the cytosol and cellular membrane of carcinoma cells, although the distribution of immunoreactivity differed among the cells in each case, indicating that the cellular distribution of LPA2 is highly heterogeneous, even within the same tumour.

The exact role played over-expression of LPA2 in breast carcinogenesis is not yet clear. However, in ovarian cancer cells the proliferative responses to LPA of ovarian cancer cells with high levels of LPA1 receptor were uniformly weaker than those of cells with low LPA1 receptor levels [22]. Moreover, LPA1 has been reported to transduce the inhibitory signal to LPA2-mediated proliferative responses and concurrently elicits apoptosis and anoikis [23]. On the other hand, LPA has been shown to have an antiapoptotic effect on various cells, although the LPA receptor that mediates this response has not been determined [24,25]. Deng and coworkers [26] reported that LPA prevents apoptosis of rat intestinal epithelial cells (IEC-6) induced by radiation and chemotherapeutic agents. In that study they used receptor specific inhibitors and concluded that the antiapoptotic effect was mediated through both LPA1 and LPA2. Thus, LPA1 can mediate both antiapoptotic and apoptotic effects, and this is apparently dependent on the cell type and circumstances. Taken together, these findings indicate that the stimulation of LPA1 receptor has suppressive effects, at least in part, on cell proliferation as compared with the LPA2 receptor. Thus, the enhanced expression of LPA2 with relatively reduced expression of LPA1 appears to promote mammary epithelial cell survival in LPA-rich circumstances and to favour development of breast cancer.

Immunohistochemical finding showed that LPA2 is upregulated more frequently in postmenopausal than in premenopausal women, suggesting that over-expression of LPA2 is more strongly related to the carcinogenesis of postmenopausal breast cancer. It is well known that a high-fat diet has a positive association with development of breast cancer, especially postmenopausal breast cancer, although some conflicting findings have also been reported [27-32]. A high-fat diet is often associated with the insulin resistance syndrome, and hyperinsulinaemia and high level of insulin-like growth factor (IGF) have also been reported to be markers of increased breast cancer risk [33-36]. Recently, receptors for IGF-1 were shown to be functionally expressed in mammary epithelium [37-39]. Moreover, the IGF-mediated intracellular signal closely interacts with the LPA-mediated signal, although the specific mechanism is not yet known [40-43]. Therefore, our data raise the possibility that a shift in LPA receptor expression may alter the effects of the LPA on IGF-mediated signal in mammary epithelial cells, which may be closely associated with adiposity-related carcinogenesis of breast cancer. The crosstalk between the LPA1- or LPA2-mediated signal and the IGF- or insulin-mediated signal remains an interesting subject for future study.

In summary, we found preferential expression of LPA2 in ductal carcinoma as compared with normal epithelium in human mammary gland tissue. Over-expression of LPA2 was more prominent in postmenopausal women. A shift in LPA receptor expression in mammary epethelium may be a key event, especially in adiposity-related breast carcinogenesis. LPA and its receptors could represent new chemopreventive targets in a novel therapeutic strategy in human breast cancer.

## Conclusion

Human ductal carcinoma cells frequently exhibited enhanced expression of LPA2 as compared with normal epithelium in human mammary gland tissue, especially in postmenopausal women. This suggests that upregulation of LPA2 may be one of the key events in carcinogenesis, especially in adiposity-related breast cancer.

## Competing interests

The authors declare that they have no competing interests.

## Abbreviations

IGF = insulin-like growth factor; LPA = lysophosphatidic acid; PBS = phosphate-buffered saline; PCR = polymerase chain reaction; RT = reverse transcription.

## Figures and Tables

**Table 1:** Lysophosphatidic acid receptor 2 (LPA2) expression evaluated by RT-PCR and immunohistochemistry

Immunohistochemistry findings	Relative mRNA expression		*P*
		
	<3000	>3000	
Low expression	10	1	
High expression	3	11	<0.001

**Table 2:** The relationship between lysophosphatidic acid receptor 2 (LPA2) expression and clinical/pathological factor

LPA2 expression	Low (36)	High (48)	*P*
Age (years)			
<50	20 (56%)	16 (33%)	0.042
≥50	16 (44%)	32 (67%)	
Tumour size			
T1 (≤2.0 cm)	16 (44%)	17 (35%)	NS
T2 (>2.0 cm to ≤5.0 cm)	18 (50%)	25 (52%)	
T3 (>5.0 cm)	2 (6%)	6 (13%)	
Nuclear grade			
I	5 (14%)	5 (10%)	NS
II	24 (67%)	31 (65%)	
III	7 (19%)	12 (25%)	
Menopausal status			
Premenopausal	21 (58%)	17 (35%)	0.037
Postmenopausal	15 (42%)	31 (65%)	
Oestrogen receptor status			
Negative	10 (55%)	27 (56%)	NS
Positive	8 (45%)	21 (44%)	
Nodal metastasis			
Negative	12 (33%)	21 (44%)	NS
Positive	24 (67%)	27 (56%)	
Distant metastasis			
Negative	27 (75%)	41 (85%)	NS
Positive	9 (25%)	7 (15%)	

Numbers in parentheses indicate percentages in the same expression pattern. NS, not significant.
